# Dilatation aigue de l'estomac: à propos de 02 cas et revue de la literature

**DOI:** 10.11604/pamj.2015.22.210.8092

**Published:** 2015-11-04

**Authors:** Alpha Oumar Touré, Ousmane Thiam, Mamadou Cisée, Diomede Nduwimana, Mohamadou Lamine Gueye, Mamadou Seck, Ousmane Ka, Madieng Dieng, Cheikh Tidiane Touré

**Affiliations:** 1 Service de Chirurgie Générale, CHU Aristide Le Dantec, Dakar, Sénégal

**Keywords:** Dilation gastrique, nécrose gastrique, rupture gastrique, gastrectomie, gastric dilatation, gastric necrosis, gastric rupture, gastrectomy

## Abstract

La dilatation aiguë de l'estomac est une pathologie rare. Elle est classiquement observée en psychiatrie dans les troubles du comportement alimentaire. Le diagnostic préopératoire est difficile et fait appel à la radiologie. La mortalité liée aux complications varie entre 80% et 100%. Nous rapportons 2cas de dilatation aiguë de l'estomac prises en charge au service de Chirurgie Générale de l'Hôpital Aristide Le Dantec de Dakar dont l'une était compliquée de nécrose et l'autre d'une rupture gastrique. Il s'agissait de 2 patients dont l'un était de sexe masculin âgé de 32 ans et l'autre de sexe féminin âgée de 36 ans. Ils étaient reçus dans un tableau de douleurs abdominales aiguës et un état de collapsus cardiovasculaire. L'examen avait retrouvé un syndrome d'irritation péritonéale chez les 2 patients. A la biologie, on notait une anémie chez tous les patients. A la radiographie de l'abdomen sans préparation, on notait un pneumopéritoine massif chez le patient et un gros niveau hydro-aérique chez la patiente. Le diagnostic préopératoire était une péritonite par perforation d'organe creux chez le patient et une occlusion intestinale aiguë chez la patiente. Après une réanimation, la laparotomie avait permis de retrouver une dilatation énorme de l'estomac avec une large rupture au niveau de la petite courbure chez le patient et une dilatation importante de l'estomac avec une nécrose du fundus chez la patiente. Une suture de la petite courbure était réalisée chez le patient et une gastrectomie atypique fundique chez la patiente. Les suites opératoires étaient marquées par un décès chez le patient au deuxième jour post-opératoire et une sténose gastrique chez la patiente nécessitant une gastrectomie totale.La dilatation aiguë de l'estomac est une pathologie rare. Son diagnostic aux urgences est difficile car les signes ne sont pas spécifiques. Les formes compliquées donnent un tableau d'abdomen chirurgical aigu. L'exploration chirurgicale pose le diagnostic. Le traitement des complications va de la suture à la gastrectomie. La mortalité dans les formes compliquées est élevée.

## Introduction

La dilatation aiguë de l′estomac est une pathologie rare. Elle est classiquement observée en psychiatrie dans les troubles du comportement alimentaire [1]. En postopératoire, elle peut être la manifestation de troubles électrolytiques de diverses causes [1]. Elle est rarement évoquée en préopératoire. Le tableau clinique n′est pas spécifique et le diagnostic nécessite un recours à l′imagerie. Ce retard diagnostique est souvent à l'origine des conséquences souvent dramatiques. La pose d′une sonde nasogastrique en urgence peut permettre d′éviter les complications qui nécessitent une prise en charge chirurgicale. La mortalité d′une dilatation gastrique aiguë compliquée d′ischémie et de perforation varie entre 80% et 100% [2]. Nous rapportons 2 cas de dilatation aiguë de l′estomac prises en charge au service de Chirurgie Générale de l′Hôpital Aristide Le Dantec de Dakar dont l′une était compliquée de nécrose et l′autre de rupture gastrique.

## Patient et observation

### Cas no 1

**Figure 1:**
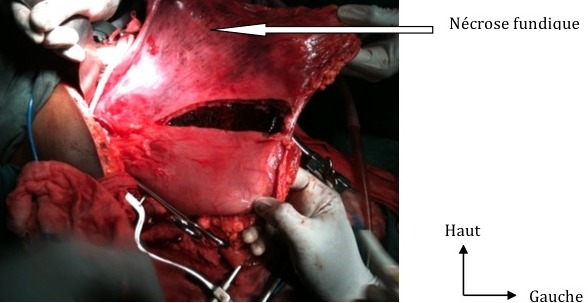
Dilatation gastrique avec une necrose du fundus

**Figure 2:**
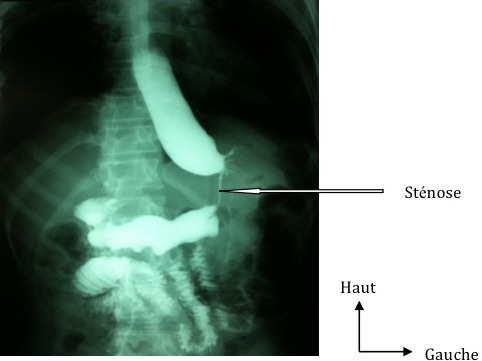
Stenose gastrique post-operatoire

Il s′agissait d′une patiente de 36 ans sans antécédents particuliers et sans troubles psychiatriques. Elle a été reçue le 20/03/2010 dans un tableau de douleurs abdominales aiguës diffuses associées à des vomissements et un arrêt du transit intestinal évoluant depuis 04 jours. A l′examen, elle présentait un état général altéré, une anémie clinique et une déshydratation. Elle était en collapsus cardiovasculaire avec un pouls filant faiblement perçu, une tension artérielle à 80/50 mmHg et une polypnée superficielle à 40 cycles/min. L′abdomen était le siège d′un météorisme asymétrique et d′une irritation péritonéale. Elle avait bénéficié d′une réanimation par la perfusion de macromolécules qui avait permis de stabiliser les constantes hémodynamiques. A la numération et formule sanguine, on avait retrouvé une anémie à 9g/dl et un taux de globules blancs à 8000 éléments/mm3. L'ionogramme sanguin avait révélé une hypokaliémie à 2,8 mmol/l et une hyponatrémie à 128 mmol/l. Il existait une insuffisance rénale fonctionnelle avec une créatininémie à 20 mg/l. Une radiographie de l′abdomen sans préparation de face avait montré un gros niveau hydro-aérique sous la coupole diaphragmatique gauche et une grisaille abdominale. Une laparotomie xypho-pubienne avait permis de retrouver un estomac très dilaté avec nécrose du fundus sans limite nette et sans obstacle duodénal (Figure 1). Il a été réalisé une gastrectomie polaire supérieure atypique par résection du fundus nécrosé et une jéjunostomie d′alimentation. Le transit oesogastroduodénal (TOGD) réalisé au 11ieme jour postopératoire avait mis évidence une sténose gastrique étendue et incomplète (Figure 2). Une gastrectomie totale avec un rétablissement de la continuité digestive par anastomose œso-jéjunale sur anse en Y de ROUX a été réalisée au 16ieme jour postopératoire. Les suites opératoires étaient simples et la patiente a été mise en exeat au 17ieme jour de sa deuxième intervention. Son devenir à moyen et long terme n′est pas connu puisqu′elle a été perdue de vue.

### Cas no 2

**Figure 3:**
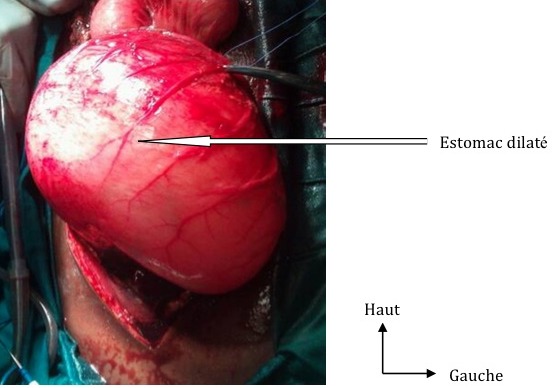
Dilatation gastrique énorme

**Figure 4:**
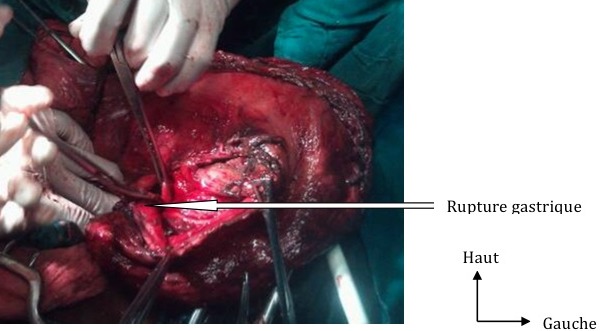
Rupture de la petite courbure gastrique

Il s′agissait d′un patient de 32 ans, sans antécédents particuliers. Il a été reçu le 06/08/2012 aux Urgences Chirurgicales pour des douleurs abdominales du flanc gauche d'installation progressive évoluant depuis 06 heures associées à des vomissements. Ces douleurs étaient consécutives à une alimentation importante suite à la rupture du jeûn durant le mois de ramadan. A l'examen, le patient était en état de choc avec une tension artérielle et un pouls imprenables, une hypothermie à 34,2°C et une polypnée à 36 cycles/min. Les muqueuses conjonctivales étaient pâles. L'abdomen était distendu avec un tympanisme diffus. Il y avait une contracture abdominale avec une crépitation gazeuse au niveau hypogastrique et du flanc gauche. Au toucher rectal, le cul de sac de Douglas était libre et indolore, le doigtier revenait souillé de sang noirâtre. La sonde nasogastrique avait ramené du sang noirâtre. La numération et formule sanguine faite à l'admission avait permis de retrouver un taux d'hémoglobine à 12,7g/dl et le reste des éléments sanguins était normal. La fonction rénale était altérée avec une créatininémie à 22,29g/l et un taux d'urée à 0,47g/l. Le bilan de la coagulation était normal. La numération de contrôle faite 5 heures après son admission avait permis de noter une chute importante du taux d'hémoglobine à 6,2g/dl. La radiographie de l'abdomen sans préparation avait objectivé un pneumopéritoine massif. Le diagnostic de péritonite par perforation d'organe creux était retenu. Une réanimation préopératoire avait permis de corriger les constantes hémodynamiques avec une tension artérielle à 115/70mmHg et un pouls à 110 pulsations/min. L'exploration par laparotomie médiane xypho-pubienne avait permis de retrouver une issue de 1,5 l de sang noirâtre mêlé à des débris alimentaires, une dilatation importante de l'estomac arrivant au pelvis et une rupture de l'estomac sur 12 cm au niveau de la petite courbure vers le cardia avec des berges saignantes (Figure 3, Figure 4). Le patient avait bénéficié d'une suture de la rupture gastrique par un surjet au polyglactin 910 (vicryl*2/0), une ligature des branches terminales de l'artère gastrique gauche qui saignaient, une toilette et un drainage avec une lame de Delbet. Il était ensuite transféré à la réanimation, intubé, ventilé, sous noradrénaline. Le décès était survenu au 2^ieme^ jour post-opératoire dans un tableau de collapsus cardiovasculaire irréversible.

## Discussion

La dilatation aiguë de l'estomac est une pathologie rare. Dans la littérature, 37 cas ont été retrouvés sur 10 ans [3, 4]. Ce sont les mêmes constats de Lee et al qui ont retrouvé une vingtaine de cas entre 1985 et 2004 [5]. Sa fréquence est passée de 1 cas par an en moyenne il y a dix ans à 3,9 cas par an actuellement d'après la littérature [6]. Si on se base sur les 37 cas retrouves dans la littérature dont le sexe n'a pas été spécifié dans 1 cas, on note 26 femmes et 10 hommes. Ceci rejoint les considérations de Aydinet al pour qui la dilatation aiguë de l'estomac concerne la femme dans 37% des cas [7]. C'est une pathologie qui peut se voir à tout âge [4, 8]. C'est une affection ubiquitaire intéressant toutes les races. Les causes sont diverses. Les causes les plus fréquentes dans la littérature sont l'anorexie mentale, l'aérophagie et la polyphagie, un repas ponctuel excessif, le syndrome de la pince aorto-mésentérique etc... [8]. Les circonstances étiologiques ne sont pas connues chez notre premier patient. Elle pourrait être secondaire à une polyphagie. Celle-ci pourrait ne pas être avouée par la patiente du fait de la discrétion culturelle des comportements alimentaires de nos populations. Chez notre deuxième patient, la rupture gastrique était survenue au décours d'une boulimie après la rupture du jeûn. Chez ce malade, le jeûn peut être assimilé à une anorexie mentale. Au cours des anorexies, il est démontré à la manométrie une diminution du péristaltisme duodénale et antrale entrainant un allongement du temps de vidange gastrique responsable de la dilatation gastrique lors d'une alimentation massive. Benson et Ward ont rapporté l'association d'une dilatation aiguë de l'estomac à une pancréatite à la suite d'une rupture de jeûn pendant le Ramadan qu'il suggère dénommer «Ramadan syndrome» [9]. Les signes fonctionnels les plus retrouvés sont des signes d'occlusion intestinale haute [5]. Ceci a été retrouvé chez un de nos patients. Les signes fonctionnels peuvent être moins marqués. Ainsi, Algiakrishnan et al ont rapporté un sexagénaire diabétique qui présentait une diarrhée et une distension gastrique indolore [4]. Les signes de choc retrouvés chez nos patients étaient aussi retrouvés par Watanabe et al dans 12/20 patients [10]. Ces signes de choc sont dus au troisième secteur, aux vomissements et à la compression de la veine cave inferieure par l'estomac dilaté [11]. La distension abdominale est constante dans la dilatation aiguë de l'estomac. Elle est souvent asymétrique prédominant à l'hypochondre gauche et l’épigastre. La nécrose de l'estomac sur dilatation aiguë est suspectée devant l'aggravation du tableau clinique et à partir des explorations radiologiques qui retrouvent une dilatation gastrique associée à une pneumatose pariétale avec ou sans aéroportie, une absence de rehaussement de la paroi gastrique au scanner après injection de contraste [12]. Comme chez notre premier cas, Lunca et al stipulent que la majorité des cas de nécrose surviennent le long de la grande courbure et sur le fundus [13]. Ce pendant, la richesse vasculaire de l'estomac l'expose moins à la nécrose. Comme chez notre deuxième patient, Adam et al ont avancé que le siège de la rupture se situe dans 80% des cas au niveau de la petite courbure sous cardiale dans une zone de fragilité appelée locus minorisresistentiae [3]. Par contre, Ourfali et al ont décrit que la rupture intéresse dans 40% la face antérieure, 23% la grande courbure et 15% la face postérieure ou la petite courbure [14]. L'image évocatrice à la radiographie de l'abdomen sans préparation est la présence d'une volumineuse poche à aire gastrique allant de l'hypochondre gauche à l'hypochondre droit. La TDM abdominopelvienne a une meilleur sensibilité pour poser le diagnostic, de retrouver les complications et la cause [13]. La réanimation médicale est la première étape de la prise en charge avec une bonne correction des troubles hydro-électrolytique surtout en cas de choc hypovolémique. La mise en place d'une sonde nasogastrique en aspiration douce associée à une correction hydro-électrolytique est l'attitude la mieux partagée [11]. La chirurgie est indiquée en cas de complication comme une nécrose, une perforation et une rupture. Dans les nécroses gastriques, une gastrectomie atypique ou non est indiquée. Chez notre patiente, on avait réalisé une gastrectomie atypique. Ce pendant, Ammori et al avait réalisé une gastrectomie totale [15]. Le traitement de la rupture gastrique reste chirurgical par une suture. Dans la littérature, on a retrouvé 1 cas de rupture gastrique qui a été traitée avec succès selon la méthode de Taylor [3]. Cette dernière se fait en milieu chirurgical et demande une disponibilité de l'imagerie diagnostique et interventionnelle, une bonne stabilité hémodynamique, en dehors de toute irritation péritonéale et à l'absence de toute défaillance viscérale. Les suites opératoires de la gastrectomie atypique étaient marquées par la survenue d'une sténose gastrique étendue. Cette sténose était probablement due à l’étendue de l'exérèse de la nécrose. Cette nécrose fungique étendue pourrait faire discuter une gastrectomie totale lors de la première intervention. Le décès de notre deuxième patient pourrait être dû au choc hémorragique et du retard de la chirurgie. Ceci impose la prise en charge précoce des ruptures gastriques du fait des lésions vasculaires qu'elle entraine souvent. La mortalité d'une dilatation aiguë de l'estomac avec rupture gastrique est très élèvée entre 50 et 65% même après chirurgie [8].

## Conclusion

La dilatation aigue de l'estomac est une urgence médico-chirurgicale rare. La clinique n'est pas spécifique. La radiographie de l'abdomen sans préparation et le scanner sont d'un grand apport dans la prise en charge. Les complications telles que la nécrose et la rupture sont redoutables. La chirurgie reste le principal traitement des complications avec une lourde mortalité.
